# An uncommon cause of early infantile liver disease and raised chitotriosidase

**DOI:** 10.1002/jmd2.12123

**Published:** 2020-04-15

**Authors:** Srividya Sreekantam, Hina Rizvi, Rachel Brown, Saikat Santra, Julian Raiman, Suresh Vijay, Patrick J. Mckiernan, Girish L. Gupte

**Affiliations:** ^1^ Department of Clinical Inherited Metabolic Disorders Birmingham Women's and Children's Hospital NHS Trust UK; ^2^ Department of Hepatology Birmingham Women's and Children's Hospital NHS Trust UK; ^3^ Department of Histopathology Birmingham Women's and Children's Hospital NHS Trust UK; ^4^ Department of Gastroenterology, Hepatology, and Nutrition Children's Hospital of Pittsburgh Pittsburgh Pennsylvania USA

**Keywords:** acute on chronic liver disease, glycogen storage disease type IV, GSD IV, raised serum chitotriosidase

## Abstract

Our subject presented at 11 months of age, following a varicella zoster infection, with acute on chronic liver disease and was found to have raised serum chitotriosidase. White cell enzyme analysis for Gaucher, Niemann Pick A, B and lysosomal acid lipase deficiency were normal. Niemann Pick type C (NPC) disease was considered as a provisional diagnosis and liver transplantation assessment deferred until recovery from varicella and results of mutational analysis of NPC gene were available. Liver biopsy at a later date showed findings suggestive of glycogen storage disease (GSD) type IV but he was too unstable for an urgent liver transplantation and sadly passed away at the age of 13 months. The classic hepatic subtype of glycogen storage disorder type IV (GSD IV) is a rare metabolic cause of early‐onset liver disease and raised chitotriosidase. There are very few reports of raised chito in GSD IV. Liver transplantation has a favourable outcome for the hepatic subtype of GSD IV and early diagnosis in our subject could have potentially altered the outcome.

## 1 INTRODUCTION

Niemann Pick disease type C (NPC), Gaucher disease and lysosomal acid lipase deficiency are well‐known metabolic causes of severe liver disease and macrophage activation in infancy. Although rare, the classic hepatic subtype of glycogen storage disorder type IV(GSD IV) is characterised by early‐onset liver disease and is unique in that liver transplantation has a favourable outcome in these patients. There are very few case reports of raised chitotriosidase activity in GSD IV.^1^, ^2^ We report a 13‐month‐old boy with GSDIV who presented with early‐onset chronic liver disease and raised chitotriosidase but was critically ill when listed for liver transplantation and sadly succumbed to fulminant hepatic failure.

**SYNOPSIS**

To consider GSD IV as a possible cause of acute on chronic liver disease with raised chitotriosidase as liver transplantation has a beneficial role in altering the outcome of those with mainly hepatic manifestations of this condition.

## 2 CASE PRESENTATION

This boy was born at term to non‐consanguineous Caucasian parents after an uneventful pregnancy. Developmental history was unremarkable. He was noted to have a distended abdomen at 6 months of age and was referred to secondary care at the age of 11 months in view of worsening abdominal distension, jaundice and lethargy following varicella zoster infection. On examination, he had icterus and mild respiratory distress. His abdomen was distended with dilated abdominal veins; liver was enlarged at 7 cm and the spleen 10 cm down the costal margin. He was found to have ascites, conjugated hyperbilirubinemia and synthetic hepatic dysfunction with coagulopathy and hypoalbuminemia. Serum bilirubin was 104 μmol/L(0‐18) with a direct component of 91 μmol/L (0‐11). Alkaline phosphatase was 1246 IU/L(90‐290), alanine transaminase and aspartate aminotransferase were 217 IU/L (0‐50) and 609 IU/L (0‐50), respectively. Clotting was deranged with a prothrombin time of 28 seconds (9‐13), APTT of 37 seconds (24‐32) and fibrinogen of 0.8 (1.5‐4.5). Albumin was 33 g/L (43‐54). He had thrombocytopenia with a platelet count of 90 (150‐400).

Renal tubular reabsorption of phosphate was normal. Urine organic acids showed raised 4‐hydroxyphenyl pyruvate and 4‐hydroxyphenyl lactate consistent with liver dysfunction. Galactosemia, tyrosinemia and mitochondrial DNA depletion screen were unremarkable. Serum chitotriosidase was elevated at 28.00 μmol/min/L (0.1‐2.5). White cell enzyme assay for Gaucher, Niemann Pick A and B and lysosomal acid lipase deficiency were all within normal limits. Bone marrow aspirate showed no evidence of infiltration.

Ultrasound scan of the abdomen showed dampened flow to hepatic veins and CT angiogram of the abdomen demonstrated a thrombus in portal vein and narrowing of hepatic veins raising the possibility of Budd‐Chiari syndrome.

NPC was considered as the provisional diagnosis in view of the clinical picture and raised serum chitotriosidase, despite the normal bone marrow biopsy. Mutation analysis was scheduled, but at the time turnaround was slow. Consideration of liver transplantation and hepatic venography with transjugular liver biopsy was postponed until he recovered from varicella zoster. Echocardiography was normal and did not show any evidence of cardiomyopathy.

### 2.1 Progress

**FIGURE 1:**
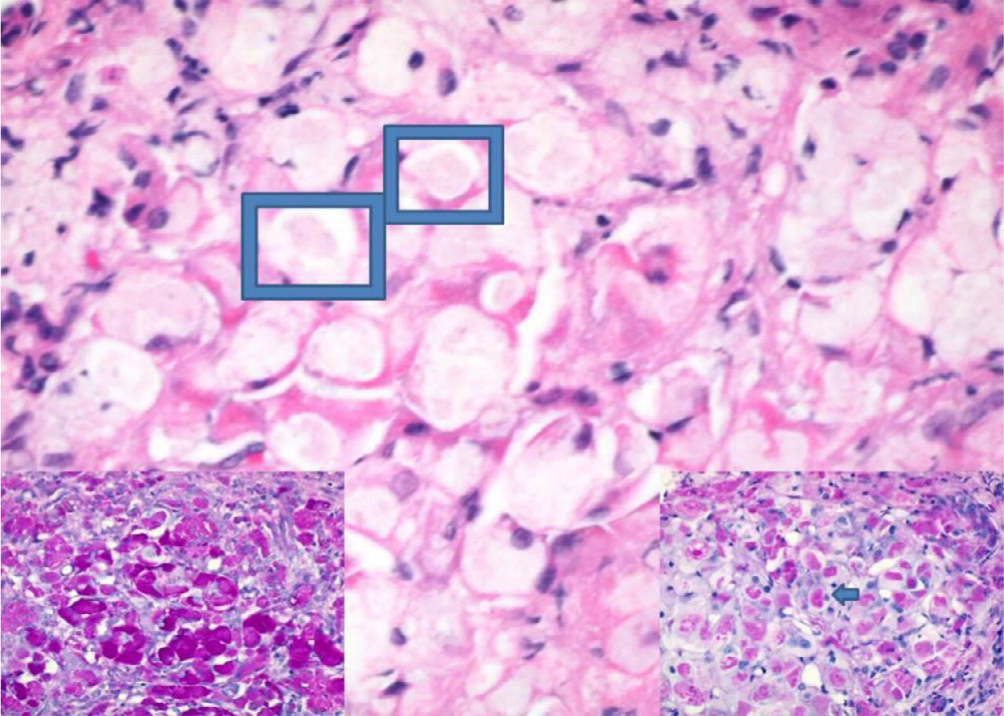
Liver biopsy shows abundant in part very large non‐membrane bound inclusions composed of fine fibrillary material admixed with more coarse glycogen. Membrane bound similar appearing inclusions are seen in macrophages. These appearances further support a diagnosis of GSD type 4. In contrast to other forms of glycogen storage disease, the hepatocytes do not show cytoplasmic clearing. The background image (original magnification ×400, haematoxylin and eosin) shows ill‐defined pale inclusions within the cells (examples within rectangles). Staining with periodic acid Schiff (PAS), bottom left panel, highlights the inclusions and these are resistant to diastase (PASD bottom right, example arrowed)

One month later he was electively admitted for assessment and management of his liver disease. A transjugular liver biopsy and hepatic venogram were performed following which he developed respiratory distress requiring intensive care management. He developed pulmonary haemorrhage and was on high‐frequency oscillatory ventilation (HFOV). Sequence analysis of NPC 1 and 2 genes did not reveal any mutations. The liver biopsy had classical changes of GSD IV (Figure 1). He was considered for liver transplantation but was too unwell due to pulmonary haemorrhage which proved fatal. Subsequent to his death, mutational analysis of the GBE 1 gene showed that he was compound heterozygous for the p.(Arg515His), c. 1544G > A mutation and the p.(Arg524*), c.1570C > T mutation in exon 12 of the gene, confirming the diagnosis of GSDIV. These mutations have been previously reported to be pathogenic.^4^

## 3 DISCUSSION

The classic hepatic form of GSD IV rapidly progresses to cirrhosis in the first 18 months of life and results in end‐stage liver disease with death due to liver failure between 3 and 5 years of age. Our patient had features of hepatic GSD IV with accelerated disease progression probably secondary to varicella zoster infection. He presented at 11 months of age with acute on chronic liver failure which rapidly progressed, leading to death at 13 months of age. He did not have a liver biopsy at presentation due to varicella zoster infection and the elevated chitotriosidase raised the suspicion of NPC disease which was important to exclude as LT is usually contraindicated. Liver transplantation was deferred until the results of mutational analysis of NPC gene (usually available after 12 weeks) were available. Current practice with more rapid turnaround of genetic analysis would have allowed both earlier confirmation of the true diagnosis and exclusion of NPC.

Hizarcioglu‐Gulsen et al reported a similar case of GSD IV who in contrast to our case presented at the age of 3.5 years in mild acute liver failure and in whom an early liver biopsy led to the diagnosis.^1^ She was also noted to have elevated plasma chitotriosidase of 1209 nmol/h/mL (reference range: 0‐90 nmol/h/mL) and two foamy cells on bone marrow aspiration. Raised chitotriosidase is a non‐specific marker and is often seen in chronic liver diseases with macrophage activation. There are two previous reports of children with glycogen storage disease (GSD) type IV who were found to have high chitotriosidase levels.^1^, ^2^GSD type IV although very rare, should be considered as a possible differential when a child presents with stigmata of chronic liver disease and raised chitotriosidase. Liver transplantation has a beneficial role and halts the disease progression particularly in those with mainly hepatic manifestations as compared to those with extrahepatic manifestations prior to transplant.^3^ Our patient had purely hepatic involvement in terms of rapidly progressive liver failure and therefore would have fit the criteria for liver transplantation had the diagnosis been made early.

## CONFLICT OF INTEREST

The authors declare no conflicts of interest.

## INFORMED CONSENT

We confirm that no patient identifiable information has been included in the manuscript. Verbal consent has been obtained from the subject's mother.

## ANIMAL RIGHTS

This article does not contain any studies with human or animal subjects performed by the any of the authors.

## References

[1] Hizarcioglu‐Gulsen H , Yuce A , Akcoren Z , et al. A rare cause of elevated chitotriosidase activity: glycogen storage disease type IV. JIMD Rep. 2014;17:63‐66. 25155778 10.1007/8904_2014_335 PMC4241209

[2] Michelakakis H , Dimitriou E , Labadaridis I . The expanding spectrum of disorders with elevated plasma chitotriosidase activity: an update. J Inherit Metab Dis. 2004;27(5):705‐706. 15669690 10.1023/b:boli.0000043025.17721.fc

[3] Matern D , Starzl TE , Arnaout W , et al. Liver transplantation for glycogen storage disease types I, III, and IV. Eur J Pediatr. 1999;158(Suppl 2):S43‐S48. 10.1007/pl00014320 PMC3006437 10603098

[4] Iijimaa H , Iwanoa R , Tanakab Y , et al. Case report analysis of GBE1 mutations via protein expression studies in glycogen storage disease type IV: A report on a non‐progressive form with a literature review. Molecular Genetics and Metabolism Reports. 2018;17:31‐37. 30228975 10.1016/j.ymgmr.2018.09.001 PMC6140619

